# Benefits and Limitations of the Record and Replay Approach for GNSS Receiver Performance Assessment in Harsh Scenarios

**DOI:** 10.3390/s18072189

**Published:** 2018-07-07

**Authors:** Calogero Cristodaro, Laura Ruotsalainen, Fabio Dovis

**Affiliations:** 1 Department of Electronics and Telecommunications, Politecnico di Torino, 10129 Torino, Italy; fabio.dovis@polito.it; 2 Finnish Geospatial Research Institute (FGI), Geodeetinrinne 2, 02430 Masala, Finland; laura.ruotsalainen@nls.fi

**Keywords:** GNSS, SDR, record and replay, GNSS receiver testing, RF front-end

## Abstract

Global navigation satellite systems play a significant role in the development of intelligent transport systems, where the estimation of the vehicle’s position is a key element. However, in strongly constrained environments such as city centers, the definition of quality metrics and the assessment of positioning performances are challenges to be addressed. Due to the variability of different urban scenarios, the modeling of the dynamics as well as the architecture of the positioning platform, which might embed other sensors and aiding means to the GNSS unit, make it hard to define unambiguous positioning metrics. Performance assessment through analytical models and simulators can be ineffective in terms of cost, complexity, and general validity and scalability of the results. This paper shows how a record and replay approach can be an efficient solution to grant fidelity to a realistic scenario. This work discusses advantages and disadvantages with emphasis on the case study of harsh scenarios. Such an approach requires proper data collections that allow the replay phase to test the GNSS-based positioning terminals. This paper presents the results obtained on a set of field tests related to different scenarios, selected as representative for the key performance indicators assessment.

## 1. Introduction

With the advent of new services for transportation systems and the implementation of autonomous vehicles, the design of accurate and reliable positioning and navigation units has become of paramount importance. The position information derived using global navigation satellite systems (GNSS) is part of almost any outdoor positioning platform. The position obtained by exploiting systems such as the Global Positioning System (GPS), Galileo, GLObalnaya NAvigatsionnaya Sputnikovaya Sistema (GLONASS), and Beidou is often integrated with a number of other sensors able to mitigate the outages of the GNSS solution and increase the accuracy in harsh environments. The requirements for the accuracy of position information provided by the positioning terminal can vary from decimeters to hundreds of meters, depending on the application. Furthermore, in transportation applications, the positioning terminal is critical in terms of safety, liability, or security, since the end user must be able to trust the obtained position solution. Integration with inertial systems is already a common and consolidated solution [1,2], but in the future, GNSS positioning will be combined with other enabling technologies supporting intelligent transport systems (ITS), namely wireless communication and information technologies at large [3]. In particular, in road environments, the next generation of cars is targeting full connectivity with other cars and infrastructure enabling the so-called vehicle-to-vehicle (V2V) and vehicle-to-Infrastructure (V2I) communications [4].

Given this picture, the GNSS receiver performance assessment in the framework of the road environment is a key point in the development process of smart transportation that exploits the ITS technology. As part of a more complex positioning unit that we define in the following as a GNSS-based positioning terminal (GBPT), the standalone GNSS performance is often hidden since only the final position is of concern to the users. However, GNSS is still an essential part of the GBPT since many other systems provide solutions that are relative to an absolute GNSS position (e.g., inertial navigation systems, INS), or are used either to refine or aid the GNSS solution, with several possible levels of hybridization. Therefore, it is important to assess the performance of GNSS standalone in such an environment, in order to be able to define a basis for the integration with other sensors and with the communication layer.

This paper addresses the problem of GBPT testing, focusing on the specific GNSS performance, for the performance evaluation in terms of the typical key performance indicators (KPIs) of the ITS sector [5]. The evaluation should be performed by means of statistical assessments, providing scenario-independent results of general validity. This is almost impossible in diverse and variable environments (e.g., urban). GNSS performance can be quite different in small and large cities, depending, for example, on the height of the surrounding buildings, on the width of the streets, and on a plethora of other environmental features.

The record and replay technique proposed in this paper is derived from the software-defined radio (SDR) paradigm [6]. It relies on raw digitized samples of the GNSS signals collected in the field that can later be re-modulated into a signal that can be fed to any commercial receiver under test. A variety of commercial devices can record and replay GNSS signals. Some of them can also record a range of additional signals, synchronised to the GNSS input, increasing the level of playback realism [7,8]. Among the several solutions that can be found in the market, the data collection system used for the work presented in this paper is capable of performing the record and replay operations of GNSS signals at low cost.

Preliminary experimental results on the use of the record and replay method for the GBPT performance assessment have been discussed in [9,10]. This paper extends the analysis of [10], and it discusses the trade-off between the parameters and provides results that validate the method. Eventually, it highlights the strengths and weaknesses behind the use of such a method for the GBPT performance assessment in the case of different operational environments.

The paper is organized as follows. After this introduction, the next section first recalls the concept of GBPT for road applications, and then it introduces the classical approaches used for its performance assessment. The following section is focused on the record and replay approach as an innovative method that can be used for the GBPT performance assessment: first an introduction about the SDR paradigm is presented, then moving to a discussion of the technical aspects and the use of such a method for the performance assessment. A comparison among the approaches that can be used for testing is presented together with the performance metrics. Obtained results from vehicular data collections and playback operations are then presented in different operational environments. Finally, the conclusions are provided.

## 2. GNSS-Based Positioning Terminal for Road Applications

**Figure 1:**
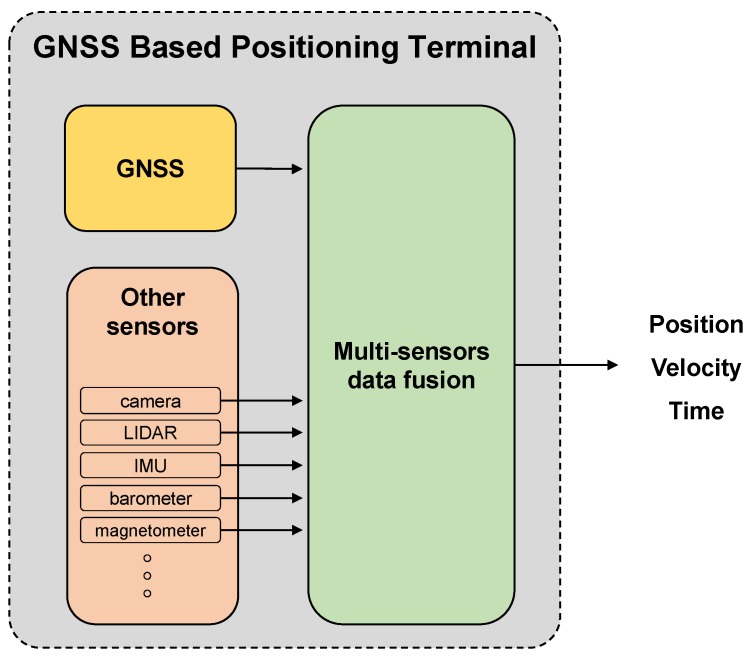
Global navigation satellite systems (GNSS)-based positioning terminal (GBPT) system architecture. IMU: inertial measurement unit.

In any road application, the knowledge of the position, velocity, and attitude of the mobile vehicle is fundamental. The availability of more GNSS satellites and constellation with respect to the past, and the increased reliability of GNSS terminals are making GNSS-based solutions the most cost-effective ones to provide an absolute reference of the vehicle with respect to a global reference frame. Such a GNSS terminal is then part of a platform where other sensors complement the weaknesses of GNSS positioning, and provide the additional information needed. The main functional components of a GBPT are depicted in Figure 1. Such a system outputs navigation data in the form of vehicle positions and velocities. These positioning metrics are delivered to the application module, and they are based on specific architectural and design implementation choices. For instance, the GBPT might be either a standalone GNSS receiver or, in most cases, a GNSS receiver hybridized with sensors according to a loose/tight coupling scheme [1].

With respect to the positioning terminal depicted in Figure 1, in this work we focus only on the performance of the GNSS module. The different sensors, which might be involved within the GBPT architecture, are thus not considered during the test phases addressed in this paper. This choice is not restrictive, since the GNSS module is the only one able to provide the absolute position of the vehicle, which is the basis for the overall positioning procedure.

## 3. Test Procedures for GBPT Performance Assessment

**Figure 2:**
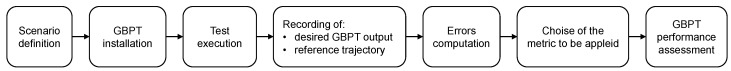
GBPT performance assessment.

As the new global navigation satellite systems and their applications are becoming available, the performance assessment of GBPT assumes considerable importance. The different approaches that are generally proposed for the performance assessment are presented hereafter. However, regardless of the chosen approach, the GBPT performance assessment flow might be represented by the main steps summarized in Figure 2. The first step is the definition of the scenario by means of trajectories and environmental conditions. Once the GBPT is installed on board a vehicle, the test can be executed and positioning data are saved. Both a reference trajectory (ground truth) as well as the desired GBPT outputs are recorded. Such data are used to compute the errors to assess the metrics that define the GBPT performance.

### 3.1. Laboratory Tests

For the laboratory tests, radio frequency constellation simulators (RFCSs) were exploited to define the scenario in a controlled and repeatable way. Several parameters, such as the pre-defined trajectory, the satellite geometry, the simulation of the errors (e.g., ionosphere, troposphere, multipath), and the power signal level, were under the user’s control. The appropriate radio frequency (RF) output was thus determined by using mathematical models. The laboratory test was performed in a dedicated suitable area, usually an anechoic chamber or via a cable directly connecting the simulator to the GNSS antenna. During the execution of the test, the desired measurements were recorded by the GBPT under test and then compared to the reference trajectory, perfectly known from the mathematical model adopted by the RFCS. This method requires a sensitive amount of resources, especially if dynamic tests have to be performed.

An overview of this approach for automotive testing is given in [11], where the authors presented a methodology to evaluate the position availability of automotive-grade GPS receivers utilizing a multichannel satellite signal simulator in a controlled laboratory environment.

### 3.2. Field Tests

This approach relied on the use of specific test vehicles for accommodating the GBPT under test as well as the reference trajectory measurement system (RTMeS). The latter was used to establish the true position referred to as the ground truth. As an example, the features and applications of the Vehicle for experimental research on trajectories (VERT) are described in detail in [12]. After the definition of the scenario in terms of trajectories and on-board equipment installations, the test could be executed and the measurements from the GBPT and from the RTMeS were recorded. These were finally compared and thus the performance assessment of the GBPT under test was performed.

### 3.3. Record and Replay Tests

The record and replay tests can be considered as a combined solution between laboratory and field tests, consisting of recording real sensor data and replaying them in the lab to evaluate the performances of the GBPT under test. They are extensively discussed in Section 4.

## 4. Test Record and Replay Approach

The record and replay approach is based on the SDR technology, which generally refers to an ensemble of hardware and software technologies and design choices that enable reconfigurable radio communication architectures. According to this approach, functional blocks which are normally hardware implemented are realized as software modules either on programmable platforms or on reconfigurable hardware.

Implementation of the GNSS receiver according to the SDR paradigm has been widely discussed (e.g., [13]), and it is now open to new implementation strategies and uses thanks to the evolution of processors and programmable hardware (e.g., cloud-GNSS) [14]. Proper standards for the data description and exchange are also being defined [15,16].

However, in this paper, we focus on the acquisition and front-end part that allows the storage of the data for future replay, rather than on efficient implementations of the full receiver architectures. The design and implementation of a generic GNSS data acquisition system based on GNSS software radio receivers is presented, for example, in [17].

**Figure 3:**
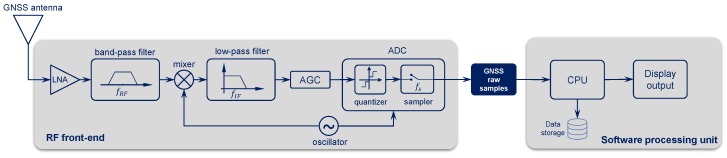
GNSS software-defined radio (SDR) data acquisition and processing system. ADC: analog-to-digital converter; AGC: automatic gain control; LNA: low noise amplifier; RF: radio frequency.

The most common architecture of a GNSS SDR receiver is composed of an antenna, a radio front-end (RFE) and a software processing unit, as shown in Figure 3.

The analog signal captured by the antenna and amplified by the low noise amplifier (LNA) is filtered to minimize out-of-band contributions and then down-converted to intermediate frequency (IF) or to baseband. An automatic gain control (AGC) can be used to automatically adjust the signal dynamics. At this point, the signal—still in its analog form—is converted into digital samples and quantized by the analog-to-digital converter (ADC). Note that the data collection process must be regulated by a proper stable clock, in order to have consistency of the collected samples. In some cases, it may be necessary to use an external reference clock to steer the clock of the analog-to-digital converter, which may not by itself grant the required stability.

The availability in the SDR chain of the signal samples, at the output of the ADC converter, allows for a recording of the GNSS signals that embed the characteristics of the environment. Such digital values are sampled at a sampling frequency $f_{s}$ and represented as binary values on a certain number of bits $n_{b}$ (typically from 1 bit up to 16 bits). This stream of digital samples is commonly denoted as GNSS raw signal or raw IF signal, and is not to be confused with the I and Q postcorrelation samples, which are the “raw” outputs of many GNSS commercial receivers. Interested readers can find more details in [18].

A proper setting of the sampling frequency $f_{s}$ and the number of bits $n_{b}$ used by the quantization process is needed to preserve the information on the specific environment, assuring the fidelity of the recorded scenario with respect to the real one. In detail, the choice of $f_{s}$ is driven by the bandwidth not only of the GNSS signal but also by other “out-of GNSS band” events that might need to be represented in the saved data log. The choice of the number of bits $n_{b}$ used by the quantization process is driven by the desired dynamic resolution of the recorded signal. However, an optimum working point between IF recording quality and data volume has to be found. If the data have to be transferred to some remote server, the required network bandwidths for the data transfer must also be considered during the design phase.

**Figure 4:**
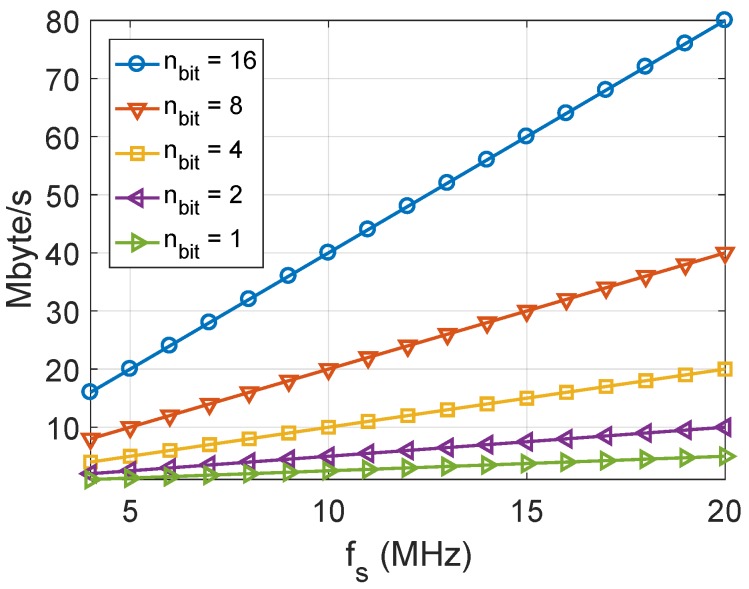
Memory requirement for a range of sampling frequencies and different quantization levels.

In the case of concatenated I and Q samples, the storage memory requirement for a range of sampling frequencies and different quantization levels is shown in Figure 4. Note that the requirement of a signal sampled by using $n_{b}$ equal to either 1, 2, or 4 would be the same as that of a signal sampled by using $n_{b}$ equal to 8, by using 1-byte coding. As an example, a data-grabber acquiring the L1 GNSS bandwidth sampled at $f_{s}=10$ MHz and $n_{b}=16$ requires 40 Mbyte/s. As a result, 30 min of raw data amount to about 72 GB.

The recording system is coupled with a dual system that implements the replay. The whole system, denoted as record and replay, has gained much attention in recent years, and the design and the implementation are available in the literature. In [19], the authors present a detailed description of the design of a system capable of replaying narrowband GNSS IF signals. They also compare the performance of a replayed data set with its live counterpart with regards to position, timing, and signal-to-noise ratio (SNR). In [20], the author focuses on the setup of the hardware components and assesses the performance of a commercial receiver in terms of signal strength and position. In [21], the design challenges of a system able to record and replay GNSS signals for multiple constellations and frequency bands are presented.

**Figure 5:**
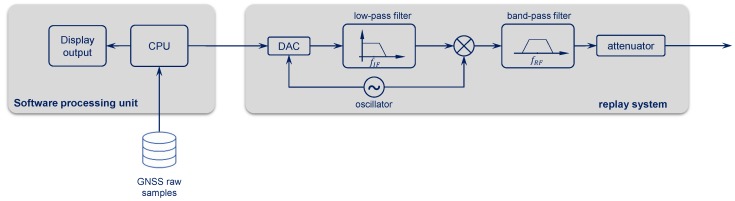
Software processing unit and GNSS replay system. DAC digital-to-analog converter.

The replay system reconstructs and modulates the signals from the recorded digital scenario at IF. Basically, the replay system works as an inverted front-end. In fact, starting from the samples, an analog signal must be created by means of a digital-to-analog converter (DAC) and filtering stage. Then, the signal can be modulated to the original RF frequency and band pass-filtered to remove the image signal. Figure 5 shows the functionality of the components within the replay chain. Depending on the power of the generated signal, an attenuation stage may be needed to emulate the power level received at the output of an active antenna. It is recommended to use a high-quality external reference clock to avoid introducing spurious components to the signal. Note that a too-small $n_{b}$ limits the fidelity of the replayed scenario, sometimes introducing artifacts in the results.

Although the storage of raw signal samples requires large storage capabilities and/or large-bandwidth data connections (in case the collected data have to be transferred), there are several advantages offered by this method for performance evaluation. It enables the possibility of recording a specific event or scenario from the real world and playing back the scenario for deeper and refined analyses, thus granting the principle of repeatability. Some specific events may be rare, and the analysis in real-time might not provide sufficient information. Nevertheless, the performance of different receiver configurations and architectures, or even different receivers, can be evaluated.

The data samples can also act as a basis for the creation of synthetic but realistic scenarios by adding impairments in a controlled environment. This approach is useful for assessing the impact of impairments such as radio frequency interference (RFI). Instead of using models that are often over-simple, the RFI can be added to the replayed signal by mixing them in a lab environment. In this case, the parameters of the interfering signals are under the user’s control, thus allowing a parametric assessment of the performance with respect to the nature and features of the interfering source that is synthetically created.

### The Use of Record and Replay for GBPT Performance Assessment

The record and replay approach for the assessment of the GBPT’s performance can be considered as the combination of laboratory and field tests. It relies on the use of a SDR front-end such as the one previously described that will act as a data grabber. The data are stored on a memory mass, and it is quite clear that the amount of data to be recorded is much larger than the output rates of the observables usually provided by GNSS receivers.

As previously remarked, testing the GNSS functionalities within a GBPT is not always straightforward, and in this section, we discuss how the record and replay approach can be helpful.

**Figure 6:**
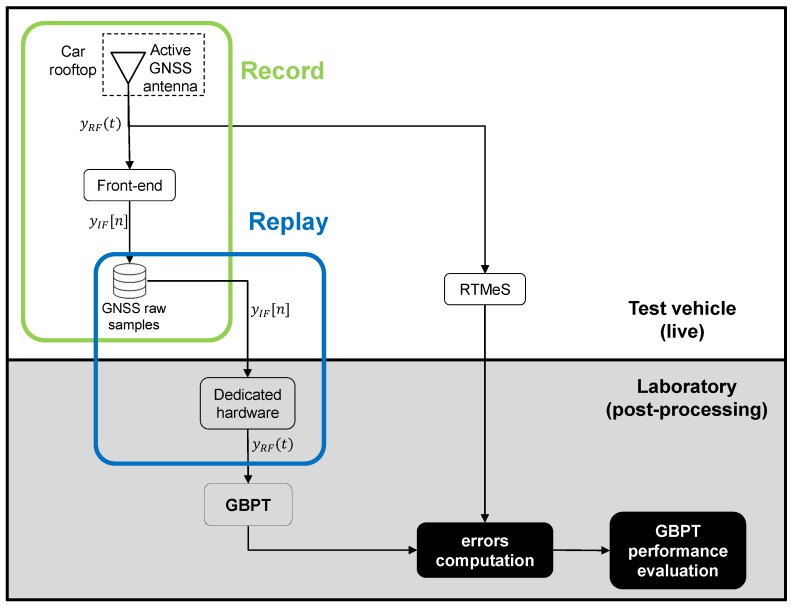
Record and replay system architecture for GBPT performance testing.

Figure 6 shows the system architecture for GBPT performance testing by using the record and replay approach. The recording and replay systems are respectively depicted as green and blue boxes. The picture also highlights which operations are expected to be live-performed on-board the test vehicle (upper part of the picture) and the ones to be carried out in the lab at a later stage (lower part of the picture). In case the output of other sensors is of interest, they need to be collected as well, and it is important to grant the time tagging of the samples by a common stable clock.

During the data collection process, the GNSS signal is split between the front-end and the RTMeS. The former records the raw samples of the GNSS signal (green box in Figure 6), whereas the latter estimates the ground truth. Starting from the samples, the re-played GNSS signal is fed into the GBPT under test (blue box in Figure 6) and the outputs of interest are saved. Output positioning data are compared to the reference trajectory, and eventually used to compute the errors for the GBPT performance assessment.

## 5. Test Procedures Comparison

A comparison of the different approaches generally proposed for the GBPT performance assessment is discussed in this section, together with the different performance metrics. Depending on the specific requirements and constraints, they present advantages and drawbacks, as summarized in Table 1. However, they all are valid tools for testing since the characteristics of one device cannot replace the functionality of another.

Among the advantages offered by the lab tests is that the user has the ability to define different scenarios. Moreover, the tests can be repeated as many times as desired under exactly the same known conditions. However, the major problem associated with such tests is that it is very difficult to model the signal degradation in the case of constrained environment scenarios such as the urban environment, so they ultimately offer low realism. Another drawback is that the hybridization of GNSS and other positioning sensors may be simulated only up to a certain level.

In contrast, field tests present high realism because they allow the investigation of conditions that are difficult to simulate. Another advantage is that they are suitable for testing hybridized GBPT. However, they present the limitation that the environments are usually time-varying and so exhibit low repeatability.

Finally, the use of the record and replay approach has the significant advantage of being very close to the real world, so it offers high realism. For this reason, it can be used for performance assessment. However, to have a stable and reliable statistic of the results, long data collections may be necessary. Furthermore, in order to obtain unbiased parameter statistics such as mean and standard deviation of the position, the results have to be averaged over a sufficient number of “consistent” scenarios. Although the use of replayed scenarios embeds the limitation that the scenario cannot be changed once the data are collected, the record and replay approach offers high repeatability. The recorded data can be replayed as many times as desired and then the signal can be fed to different devices under test to assess their performance in the desired scenario. However, as in the case of the lab tests, this approach exhibits some limitations when hybridized GPBTs are concerned. It is not straightforward to synchronously replay the GNSS signal and the other signals coming from other sensors in the lab. However, in some cases (e.g., if hybrid GNSS solutions have to be tested), the time series of the measurement of the other sensors do not need to be replayed, and only the GNSS scenario can be modified (e.g., adding interference), thus testing the robustness of the hybrid receiver. Nevertheless, it is important to remark that this approach is valuable if the core structures of the data collection system do not mask or affect the meaningful features of the collected signals, thus preserving the information on the specific environment.

## 6. Performance Metrics for GBPTs

The performance of the GBPT can be characterized with respect to different features, quantified by a corresponding metric. In turn, each performance feature of the terminal is quantified by a corresponding metric. In the road application domain, the most relevant performance features are availability, accuracy, and integrity.

Availability is the percentage of time during which the system can be used for the required function in a given scenario [5]. An example of a relevant metric for the availability feature is the number of epochs with a position output divided by the total number of epochs for a given operational scenario.

The accuracy can be measured by the error between the position provided by the positioning terminal, when this position is available, and the user’s “true” position, generally estimated by a reference measurement system. This error, which is a random variable, is fully characterized by its cumulative distribution function (CDF). In 2D, the error is called horizontal position error (HPE). Some relevant metrics for HPE are the 50th, 75th, and 95th percentiles.

The integrity is a measurement of the confidence the user can have in the position supplied by the system. For civil aviation, it is expressed in the form of a probability (or risk) of failure over the period during which the positioning service is provided [5]. However, the applicability of the aviation-born integrity to other transportation fields is not straightforward due to the limitation of the urban contexts. Some “local integrity” concepts, suitable to automotive applications in urban scenarios, have already been proposed [22].

A summary of the metrics for the performance characterization of the positioning terminal was proposed by the CEN-CENELEC standardization organization to [23]. However, in this work the statistical assessment of the GBPT performance evaluation was carried out by considering the horizontal accuracy only (i.e., HPE), defined as follows:

(1) $$HPE=\sqrt{{\left(x_{East}^{rx}-x_{East}^{ref}\right)}^{2}+{\left(y_{North}^{rx}-y_{North}^{ref}\right)}^{2}},$$

where:

- $x_{East}^{rx}$ and $x_{East}^{ref}$ are the east coordinates estimated respectively by the receiver under test and the reference receiver, at a specific time instant;
- $y_{North}^{rx}$ and $y_{North}^{ref}$ are the north coordinates estimated respectively by the receiver under test and the reference receiver, at a specific time instant.

## 7. Experimental Results

This section reports the results obtained by exploiting the record and replay approach for assessing the performance of the GBPT, which in this case embeds a consumer-grade GNSS receiver. Data were recorded from vehicular data collections by following a qualitative classification between urban and suburban environments, among the generic user environments described in [24]. The advantages and disadvantages of the use of the record and replay approach are highlighted for these two different operational environments.

### 7.1. System Setup

**Figure 7:**
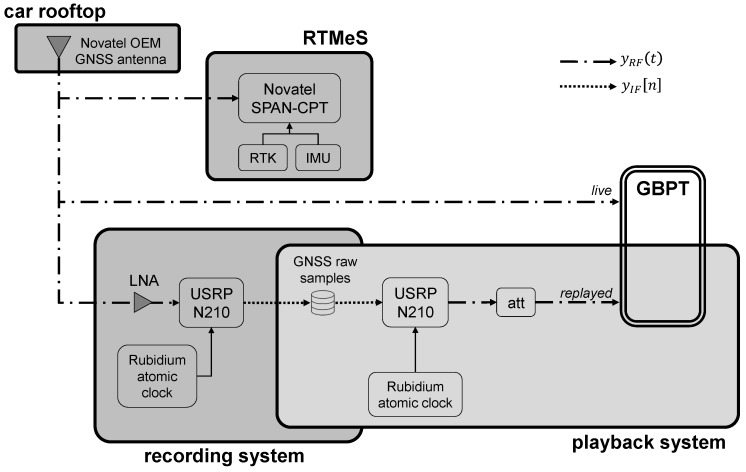
Record and replay system setup. RTMeS: Reference Trajectory Measurement System; RTK: real-time kinematic.

The setup of the record and replay system is shown in Figure 7. During the live operations performed on-board the test vehicle, the GNSS signal was first captured by the active Novatel OEM GNSS antenna (placed on the vehicle rooftop), and thus it was split among three branches. The power supply for the recording system was provided by an external battery whereas the reference receiver was powered directly from the car battery. On the other side, the playback operations were carried out in the laboratory in a post-processing stage.

The first branch included an RTMeS given by the dual frequency Novatel SPAN-CPT system receiver [25]. It is a compact, single-enclosure GNSS receiver with a tactical-grade Honeywell HG1700 IMU. The final accuracy was improved by exploiting the real-time kinematic (RTK) corrections download from the FinnRef network. FinnRef is a nationwide network of permanent GNSS stations in Finland, providing publicly available differential GNSS corrections, but also RTK corrections for scientific use.

As depicted in Figure 7, the second branch included the GBPT under test, which was a consumer-grade GNSS receiver—namely, a uBlox M8T [26].

The third branch was the recording system. Within this block, the GNSS signal was first amplified by the LNA, which provided a 30 dB gain, and then it was fed to the front-end, a USRP N210 [27]. The latter was synchronized to a Rubidium frequency standard to control the ADC in order to have a very accurate and stable sampling frequency. The modular approach of the USRP makes this front-end extremely versatile and flexible in terms of configuration parameters. Considering a trade-off between signal quality and available data storage resources, the USRP was configured by using the parameters listed in Table 2. By using these configuration parameters, 60 min of raw data amounted to approximately 72 GB.

The playback system is shown in the right part of Figure 7. It depicts the setup used in the laboratory to play back the binary samples stored on the disk, recorded during the data collections. The front-end used to reproduce the RF signal was the USRP N210, which was the same as the one used for recording the data setting the configuration parameters, listed in Table 2. It first converted the recorded samples to analog through the DAC. After a low-pass filtering stage, the samples were converted back to RF, and finally, band-pass filtered. The RF signal was attenuated to emulate the power received at the output of an active antenna and thus it was fed to the GBPT under test.

### 7.2. Analysis of the Recorded GNSS Raw Samples

**Figure 8:**
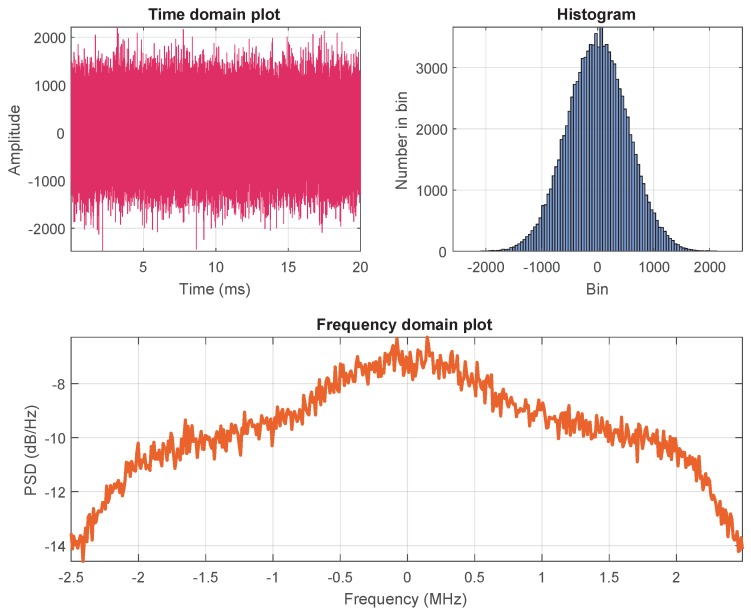
Analysis of the collected GNSS raw samples in time domain (**top**) and frequency domain (**bottom**). PSD: power spectral density.

In order to study the dynamic range of the recorded signal, the GNSS raw samples were analyzed in the time/frequency domain prior to being fed to the playback system. The analysis in the time domain is shown in the top panel of Figure 8, where the power spectral density (PSD) of the signal is shown in the bottom panel. Since the AGC was not present within the front-end architecture, the gain provided by the front-end itself was manually adjusted in order to trigger 12 bits of the ADC, out of its maximum resolution of 14 bits. The histogram, highlighting the Gaussian shape of the bins, shows its distribution over 12 bits where the output values were placed between −2048 and +2048. This design choice was twofold: on the one hand, it allows the recorded signal to be represented with a very high resolution, which might be needed to catch all the features of constrained environments. On the other hand, in the case of signal impairments such as RFI which requires higher signal power levels, it gives the possibility of enlarging the dynamic of the signal, as 2 bits are still available within the ADC. Note that this design choice also has an important role in the playback operations—namely when the recorded signal is converted back to RF and fed to a GNSS receiver, provided that the GNSS receivers are designed to receive signals within a certain power level.

### 7.3. Performance Assessment

**Figure 9:**
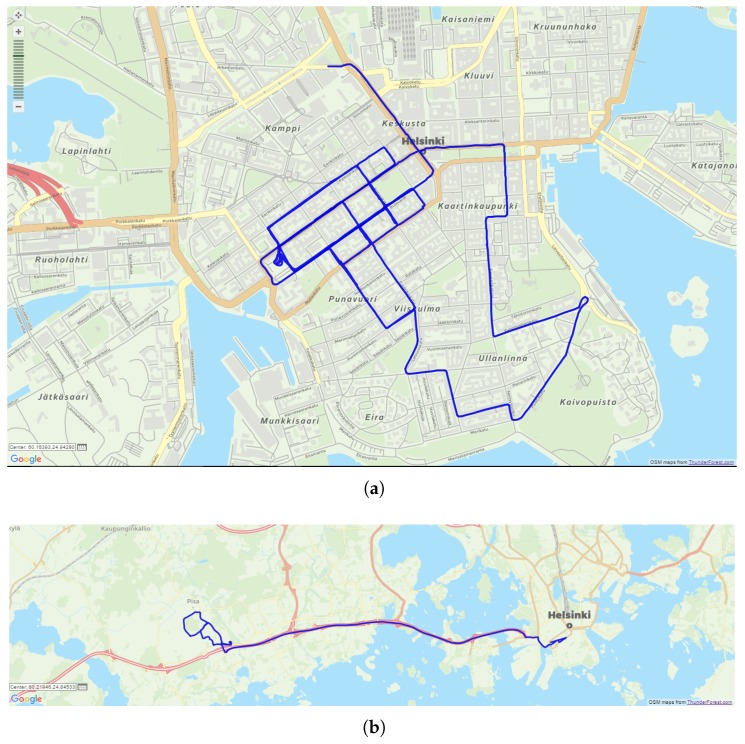
Datasets shown in Google Maps. (**a**) Urban and (**b**) suburban environments.

Data were collected within the city center of Helsinki and its suburban areas, as shown in Figure 9. In particular, the dataset related to the urban environment (shown in Figure 9a) has a duration of approximately 4200 s collected at 1 Hz, and a path length of about 20 km. The dataset related to the suburban environment (shown in Figure 9b) has a duration of about 1000 s collected at 1 Hz rate, and a path length of about 21 km.

**Figure 10:**
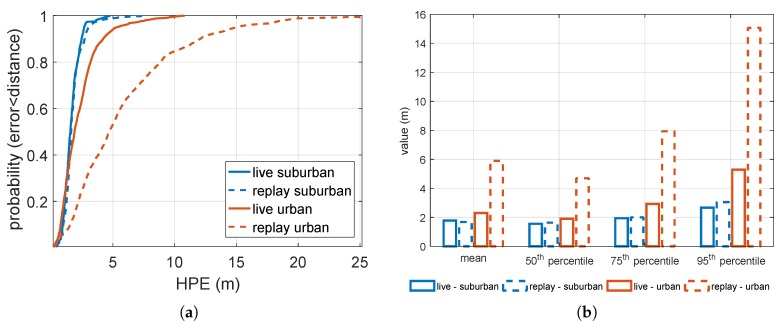
Statistical characterization of the HPE. (**a**) Cumulative distribution functions (CDFs) and (**b**) additional metrics.

The statistical characterization of the HPE related to the live and replayed trajectories is plotted in Figure 10a,b, respectively, by the continuous and dashed lines. Moreover, the blue and orange lines are related respectively to the suburban and urban environments. The CDFs of the HPE are plotted in Figure 10a.

The 50th, 75th, 95th percentiles, and the mean value of the HPEs are plotted in Figure 10b. As expected, the error on the final accuracy was larger in the case of harsh scenarios, as can be seen in the plot by comparing the continuous blue and orange lines. Such environments present several challenges to GNSS signal reception, such as blockage and reflection of the signals by buildings or trees. On the other hand, the comparison between continuous and dashed curves, which states the fidelity of the reproduced environment with respect to the real one, led to different results in suburban and urban environments. Considering for example the 95th percentile as a metric, in suburban environments they exhibited a difference of about 40 cm. This can be acceptable since it might be due to some additional noise introduced during the replay operation. On the other hand, in the urban environment they exhibited a difference of about 10 m. One of the causes of such a fidelity loss could be the signal phase noise induced by the sampling and down/up converting reference oscillator, which impacts twice in the record and replay chain, and has a larger impact in the case of the harsh urban environment with respect to the suburban one. This issue is discussed further in Section 7.4.

### 7.4. Analysis of the Discrepancies for the Urban Environment Case

The performance of the positioning terminal, denoted as GBPT in Figure 7, was assessed in the previous section by exploiting the record and replay approach. The discrepancy between the recorded and the replayed trajectories in the urban environment is further investigated in this section, since the storage of the raw signal samples enables the possibility of a deeper analysis of the signals. Among the data collected during the test campaign presented in Figure 9, a dataset captured in the city center of Helsinki was chosen as a test case. It has a duration of about 600 s.

**Figure 11:**
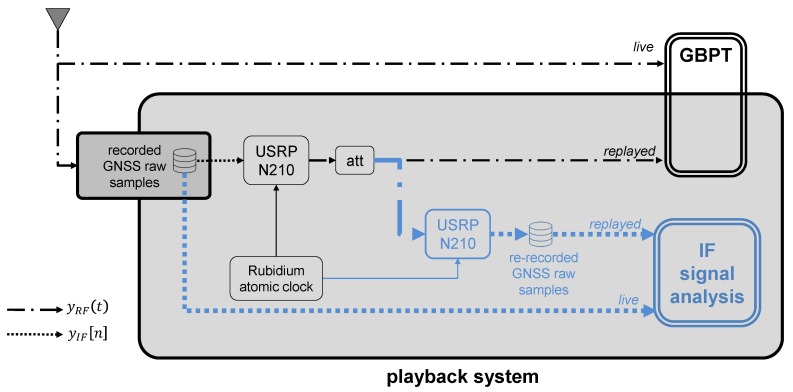
Test case for the urban environment. System setup.

In order to assess the fidelity of the live signal with respect to the one generated by the playback system, they were analyzed and compared by means of the IF raw samples. Essentially, the RF signal generated by the playback system was down-converted to IF and the raw samples were eventually saved. The system setup is shown in Figure 11.

**Figure 12:**
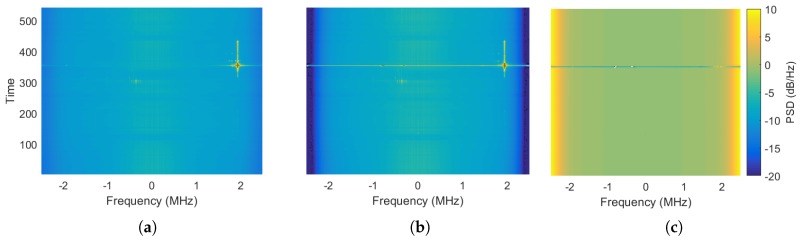
Test case for the urban environment. Spectrogram of the (**a**) recorded and (**b**) re-recorded GNSS raw samples. (**c**) Difference between the two spectrograms.

As a first analysis, the two signals were analyzed in the time–frequency domain. The spectrogram of the recorded GNSS raw samples is shown in Figure 12a, where the power spectral density is color-coded. It highlights the presence of strong interference components located at about +2 and −0.4 MHz with respect to the central frequency. In order to evaluate how the recording and playback systems behaved under such strong impairments, the spectrogram of the re-recorded GNSS raw samples was computed, as shown in Figure 12b. The difference between the two spectrograms, shown in Figure 12c, states that the two signals had the same time–frequency components, meaning that the playback system faithfully reproduced the RF signal. This was true except for the highest frequencies attenuated by the front-end filter, as well as in the time interval from 352 to 355 s.

**Figure 13:**
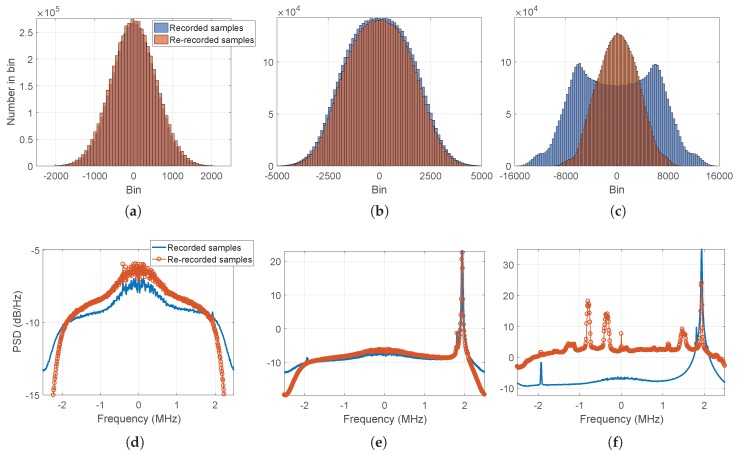
Test case for the urban environment. Histograms (**top**) and PSDs (**bottom**) of the recorded and re-recorded GNSS raw samples at seconds 155 (**left**), 351 (**middle**), and 353 (**right**).

In order to investigate this mismatch, a snapshot of the two signal samples was taken at three different time instants. The histogram and the PSD are shown, respectively, in the top and bottom panels of Figure 13 for the recorded (blue) and re-recorded (orange) signal samples. It is possible to distinguish three cases, as follows:

- Case I, Figure 13a,d: nominal conditions and faithful RF signal reconstruction. Histogram (a) and PSD (d) of the GNSS raw samples were computed at second 155. We can define this situation as nominal conditions due to the absence of interference components within the signal. Therefore, in nominal conditions the histogram of the live and replayed signal samples had the same Gaussian shape. In addition, the two signals exhibited identical spectra.
- Case II, Figure 13b,e: presence of RFI and faithful RF signal reconstruction. Histogram (b) and PSD (e) of the GNSS raw samples were computed at second 351. In that time instant, some interference components were present and located at approximately 2 MHz away from the central frequency. In correspondence of these points, the histogram of the signal samples did not have a Gaussian shape, as it should be in nominal conditions. However, the live and replayed histograms had the same shape. In addition, the two signals exhibited identical spectra, showing the capability of the system to collect and replay the full spectral information, even in non-nominal cases.
- Case III, Figure 13c,f: presence of RFI and wrong RF signal reconstruction. Histogram (c) and PSD (f) of the GNSS raw samples were computed at second 353. In that time instant, some strong interference components, located approximately 2 MHz away from the central frequency, threatened the data collection system, since the front-end went into saturation.

As stated by the analysis of the live and replayed signals at IF, the playback system was able to faithfully re-generate the RF signal except for the time interval when the front-end went into saturation. Therefore, by feeding the two signals to the GBPT under test and comparing the HPEs, one would expect a similar behavior. In other words, the two curves should match for all the signal durations except for the time interval when the front-end went into saturation, which in this specific case was from 352 to 355 s.

**Figure 14:**
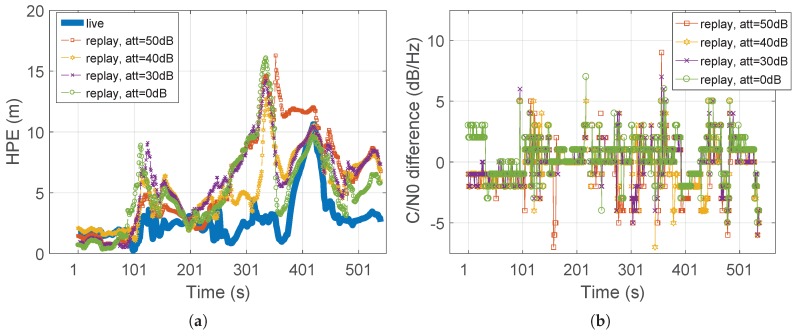
Test case for the urban environment. Different attenuation levels for the replayed signal. (**a**) HPE over time. (**b**) $C/N_{0}$ difference between live and replay signals for PRN22.

The experiment was conducted by using different levels of attenuation for the re-generated RF signal before feeding it to the GBPT. The resulting HPEs are plotted in Figure 14a. Regardless of the different attenuation levels, the HPE of the replayed signal (dashed curve) was larger than the HPE of the live signal (solid curve). As a summary, it is possible to state that in constrained environments, despite the recorded and replayed signals having the same time and frequency behavior, they did not provide the same HPE when fed to the GBPT under test. In other words, in such harsh scenarios, the record and replay system was not capable of fully reproducing the detailed recorded environment, despite the good matching of the time and frequency representations. This is also visible from the $C/N_{0}$ estimation in Figure 14b, which shows the difference of the $C/N_{0}$ values between the live and replayed signals, for all the different levels of attenuation.

The receiver reacted in a different way to the recorded data with respect to the live operation. It has to be remarked that in harsh environments the receiver is forced to react to the variability of the environment, performing a number of operations for the management of the channels, the re-acquisition of the signals, and the logic for the allocation of the resources. Such operations are based on the monitoring of some parameters and according to a rationale that is unknown to the user. The values of the monitored unknown parameters may be slightly different in the replayed signal with respect to the real case, causing the receiver to behave in a different way when the signal is far from the nominal conditions (e.g., presence of interference, distortion of the Gaussian statistics, etc.), as shown in Figure 13.

The results show that the record and replay approach should be used carefully when the recorded signal is far from the GNSS nominal condition, and it is hard to check the fidelity of the replayed signal in terms of metrics that take into account features that are important drivers for the receiver logic, but are unknown to the testing user who treats the receiver as a “black box”.

## 8. Conclusions

This paper presents some contributions to the development of a methodology for GBPT performance assessment in road applications, by exploiting the record and replay concept. The paper shows that this approach can be an efficient solution to grant fidelity to a realistic scenario. The benefits and limitations of this approach were discussed and compared to the traditional test procedures. The performance assessment of the so-called GBPT, embedding a consumer-grade GNSS receiver, was evaluated in different operational environments, such as suburban and urban scenarios. Although the obtained results are promising, different considerations must be taken into account for the considered operational environments. In particular, the results showed that the record and replay approach should be carefully used when the recorded signal is far from the GNSS nominal condition. In those cases, it is difficult to check the fidelity of the replayed signal in terms of metrics that are unknown to the testing user who treats the receiver as a “black box”. On the other hand, in less constrained scenarios, the receiver showed the same performance by processing the live and the replayed signals. The results are worthy of deeper investigation and exploitation, especially in the road transport domain.

## Figures and Tables

**Table 1:** Advantages and drawbacks of the approaches for GBPT testing. R&R: record and replay.

Approach	Cost	Realism	Complexity	Repeatability	Valid for Hybrid
Lab tests	low	low	medium	high	partially
Field tests	high	high	high	low	yes
R&R tests	medium	high	medium	high	yes

**Table 2:** USRP N210 configuration parameters.

	$f_{\mathrm{if}}$	$f_{s}$	Sampling Type	Quantization	Interface	Reference
Configuration	0 Hz (baseband)	5 MHz	I and Q	16 bits	Ethernet	Rubidium

## References

[1] Groves P.D. (2013). Principles of GNSS, Inertial, and Multisensor Integrated Navigation System.

[2] Falco G., Pini M., Marucco G. (2017). Loose and Tight GNSS/INS Integrations: Comparison of Performance Assessed in Real Urban Scenarios. Sensors.

[3] COST Action TU1302 (2015). SaPPART White Paper-Better Use of Global Navigation Satellite Systems for Safer and Greener Transport.

[4] Dey K., Rayamajhi A., Chowdhury M., Bhavsar P., Martin J. (2016). Loose and Tight GNSS/INS Integrations: Vehicle-to-vehicle (V2V) and vehicle-to-infrastructure (V2I) communication in a heterogeneous wireless network—Performance evaluation. Transp. Res. C Emerg. Technol..

[5] Peyret F. Standardization of performances of GNSS-based positioning terminals for ITS applications at CEN/CENELEC/TC5. Proceedings of the 20th World Congress on Intelligent Transportation Systems.

[6] Lo Presti L., Falletti E., Nicola M., Troglia Gamba M. Software defined radio technology for GNSS receivers. Proceedings of the 2014 IEEE Metrology for Aerospace (MetroAeroSpace).

[7] LabSat 3 Wideband. https://www.labsat.co.uk/index.php/en/products/labsat-3-wideband.

[8] Spirent GSS6425. https://www.spirent.com/Products/GSS6425.

[9] Cristodaro C., Dovis F., Ruotsalainen L. The record and replay approach for GNSS receiver performance assessment in road environment. Proceedings of the 2017 International Technical Meeting of the Institute of Navigation.

[10] Cristodaro C., Dovis F., Falco G., Pini M. GNSS receiver performance in urban environment: Challenges and test approaches for automotive applications. Proceedings of the 2017 International Conference of Electrical and Electronic Technologies for Automotive.

[11] Aloi D.N., Alsliety M., Akos D. (2007). A methodology for the evaluation of a GPS receiver performance in telematics applications. IEEE Trans. Instrum. Meas..

[12] Ortiz M., Peyret F., Renaudin V., Betaille D. (2013). From Lab to Road Test: Using a reference vehicle for solving GNSS localization challenges. Inside GNSS.

[13] Chu T., Vinande E., Akos D., Weinstein B. (2010). GNSS receiver evaluation record-and-playback test methods. GPS World.

[14] Curran J., Fernández-Prades C., Morrison A., Bavaro M. (2018). Innovation: The continued evolution of the GNSS Software-Defined Radio. GPS World.

[15] Gunawardena S., Pany T. GNSS SDR Metadata Standard Working Group Report. Proceedings of the 28th International Technical Meeting of The Satellite Division of the Institute of Navigation (ION GNSS+ 2015).

[16] Favenza A., Linty N., Dovis F. Exploiting standardized metadata for GNSS SDR remote processing: A case study. Proceedings of the 29th International Technical Meeting of The Satellite Division of the Institute of Navigation (ION GNSS+ 2016).

[17] Cristodaro C., Dovis F., Linty N., Romero R. (2018). Design of a Configurable Monitoring Station for Scintillations by Means of a GNSS Software Radio Receiver. IEEE Geosci. Remote Sens. Lett..

[18] Borre K., Akos D.M., Bertelsen N., Rinder P., Jensen S.H. (2007). A Software-Defined GPS and Galileo Receiver: A Single-Frequency Approach.

[19] Backen S., Akos D., Wilson S. (2011). RF replay system for narrowband GNSS IF signals. IEEE Trans. Aerosp. Electron. Syst..

[20] Hall D.A. (2010). Record, Replay, Rewind. GPS World.

[21] Hickling S., Haddrell T. Recording and replay of GNSS RF signals for multiple constellations and frequency bands. Proceedings of the 26th International Technical Meeting of The Satellite Division of the Institute of Navigation (ION GNSS+ 2013).

[22] Margaria D., Falletti E. (2016). The Local Integrity Approach for Urban Contexts: Definition and Vehicular Experimental Assessment. Sensors.

[23] European Committee for Standardization—European Committee for Electrotechnical Standardization (CEN-CENELEC) EN 16803-1 (2016). Space—Use of GNSS-Based Positioning for Road Intelligent Transport Systems (ITS). Part 1: Definitions and System Engineering Procedures for the Establishment and Assessment of Performances.

[24] ETSI TS 103 246-5: Satellite Earth Stations and Systems (SES), GNSS Based Location Systems, Performance Test Specification. http://www.etsi.org/deliver/etsi_ts/103200_103299/10324605/01.01.01_60/ts_10324605v010101p.pdf.

[25] Novatel SPAN Inertial Navigation System. https://www.novatel.com/products/span-gnss-inertial-systems/.

[26] Ublox NEO/LEA-M8T. https://www.u-blox.com/en/product/neolea-m8t.

[27] USRP N-210. https://www.ettus.com/product/details/UN210-KITt.

[28] Satellite Positioning Performance Assessment for Road Transport (SaPPART). http://www.sappart.net.

